# How to Clean a Mixing Slab

**Published:** 1891-04

**Authors:** 


### How to Clean a Mixing-Slab.

One of two methods may be pursued to clean a mixing-slab for cements.

Peroxide of Hyrdogen, a few drops, allowed to remain on the slab will in a short time render cleaning the surplus cement off easy.

Another way is to use strong aqua ammonia which will also readily break up the mass of cement.

From these two practical points we might draw lessons. Peroxide of oxgen should not be used more than necessary in the mouth near oxy-phosphate fillings. We infer that it is not the sulphuric acid in the peroxide that does the damage but the chemical affinity of the oxygen for the phosphoric acid. Then again the air we breath may contain ammonia, which as we have shown is a solvant of the oxy-phosphate, but according to Grouven, for every 100 lbs. body weight, an adult man expires in the twenty-fonr hours about 3.9 grains, and a boy about 6.2 grains ammonia. According to Lossen, however, the average in twenty-four hours for an adult is 0.014 grain ( 0.22 grain ). From this we see that young individuals produce more ammonia than old and small proportionately more than large animals. This may be suggestive of other ideas. W.

For Local Anæsthesia.—At the Philadelphia Hospital, local anæsthesia for minor operations is obtained by combining ten parts of chloroform, fifteen of ether, and one part of menthol, and using the mixture in a hand atomizer. After one minute's application of the spray, such a degree of anæthesia is produced that incisions can be made for the removal of growths, opening a felon or an abscess, without causing pain.

In the May Number of the Register will be given a list of the graduates from the various Dental Colleges of this country, so far at least as the commencements may have taken place. Probably a larger number of graduates, in the aggregate, than in any former year; and next year will probably show a still larger number. T.

